# The epidemiology of lymphatic filariasis in Ghana, explained by the possible existence of two strains of *Wuchereria bancrofti*

**DOI:** 10.11604/pamj.2014.17.133.3370

**Published:** 2014-02-26

**Authors:** Dziedzom Komi de Souza, Jewelna Osei-Poku, Julia Blum, Helena Baidoo, Charles Addoquaye Brown, Bernard Walter Lawson, Michael David Wilson, Moses John Bockarie, Daniel Adjei Boakye

**Affiliations:** 1 Parasitology Department, Noguchi Memorial Institute for Medical Research, University of Ghana, Legon-Ghana; 2 Yale College, Yale University, New Haven, Connecticut, 06510, USA; 3 Department of Theoretical and Applied Biology, Kwame Nkrumah University of Science and Technology, Kumasi, Ghana; 4 Centre for Neglected Tropical Diseases, Liverpool School of Tropical Medicine, Liverpool-UK

**Keywords:** Wuchereria bancrofti, genetic diversity, Lymphatic Filariasis, Ghana

## Abstract

**Introduction:**

Lymphatic filariasis is a debilitating disease caused by the filarial worm *Wuchereria bancrofti*. It is earmarked for elimination by the year 2020 through the Global Program for the Elimination of LF (GPELF). In Ghana, mass treatment has been on-going since the year 2000. Earlier studies have revealed differing epidemiology of LF in the North and South of Ghana. This study was therefore aimed at understanding the possible impacts of *W. bancrofti* diversity on the epidemiology and control of LF in Ghana.

**Methods:**

The Mitochondrial, Cytochrome C Oxidase I gene of *W. bancrofti* samples was sequenced and analyzed. The test sequences were grouped into infrapopulations, and pairwise differences (π) and mutation rates (θ) were computed. The amount of variance within and among populations was also computed using the AMOVA. The evolutionary history was inferred using the Maximum Parsimony method.

**Results:**

Seven samples from the South and 15 samples from the North were sequenced, and submitted to GenBank with accession numbers GQ479497- GQ479518. The results revealed higher mutation frequencies in the southern population, compared to the northern population. Haplotype analyses revealed a total of 11 haplotypes (Hap) in all the 22 DNA sequences, with high genetic variation and polymorphisms within the southern samples.

**Conclusion:**

This study showed that there is considerable genetic variability within *W. bancrofti* populations in Ghana, differences that might explain the observed epidemiology of LF. Further studies are however required for an in-depth understanding of LF epidemiology and control.

## Introduction

The geographic strains of parasites are important in disease pathogenicity as observed with the forest and savanna strains of *Onchocerca volvulus* [1]. Thus, an understanding of the way in which genotype varies with geography, may provide useful tools in discerning epidemiological patterns and thus designing strategies to prevent disease. Lymphatic Filariasis (LF) is a disease transmitted by the filarial worm *Wuchereria bancrofti*. It is earmarked for elimination by the year 2020, and since the year 2000 there have been yearly mass treatment of endemic communities with Ivermectin and Albendazole. Mass Drug Administration (MDA) coverage for LF increased from three million people treated in 12 countries in 2000, to nearly 950 million in 53 countries in 2011 [2]. In order to prevent failures of the massive control programs that have been embarked upon, an understanding of how genetic variations within *W. bancrofti* affect the epidemiology of LF is required in different settings.

In Ghana, studies on LF have shown differences in disease prevalence and multiplicity of symptoms in two geographically distinct regions [3]: the Northern regions of the country exhibit higher prevalence compared to the Southern regions [3, 4]. The pattern of the disease manifestations reveal a preponderance of elephantiasis (1.7%), microfilaremia (11.3%), hydrocele (20.3%) and breast lymphedema (6.6%) in the North of Ghana, compared to the south (0.3%, 0.6%, 5.2% and 6.1% respectively) [3]. While the reasons for these differences are unknown, it is could be due to the existence of different parasite strains in the country.

Few studies have pointed to genetic and morphological variations in *W. bancrofti* populations. Two different genetic variants of the parasite, have been reported, with high genetic divergence and gene flow in different geoclimatic regions in India [5, 6]. In view of the current elimination program [7], which assumes no differences within the parasite population in West Africa, further studies are required in order to formulate appropriate strategies should there be genetic variability in *W. bancrofti* that may affect the success of MDA programs. This study reports on initial findings of genetically different *W. bancrofti* strains in Ghana.

## Methods

**Figure 1:**
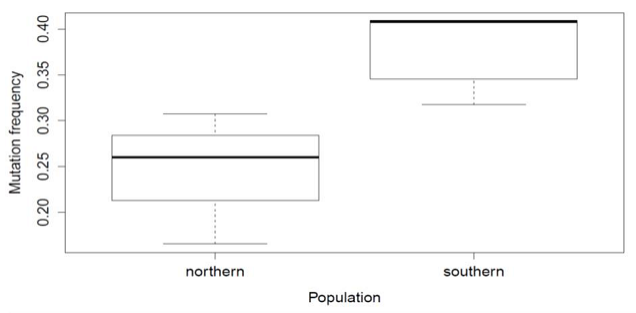
Mutation frequency plots for northern and southern population clusters.

To study the *W. bancrofti* populations in Ghana, sequence variations in the mitochondrial (mt) gene Cytochrome C Oxidase Subunit I (COI) were analyzed. The COI and MtDNA have been useful in differentiating closely related species, and as a marker for taxonomic and population genetic studies [8–10]. *W. bancrofti*were dissected out from infective *Anopheles* mosquitoes collected during two entomological studies conducted independently in the Bongo[11] and the Gomoa districts (Figure 1). The two districts are separated by a distance of about 600km, and are located in distinct ecological zones. The Bongo District is found in the Sudan Savannah while the Gomoa District is in the Guinea Savannah.

Parasite genomic DNA was extracted using the DNeasy Tissue Kit following the manufacturer's protocol (QIAGEN Inc., USA). Molecular identification of *W. bancrofti* was done as described by Ramzy and colleagues [12]. For sequencing, the COI region was amplified using two oligonucleotide primers LCO1490 and HCO2198 [13]. All PCR products were subjected to dye terminator cycle sequencing reactions and sequenced on an ABI 3730 automated sequencer, using Big Dye v3.1 and LCO1490 primer. The sequences obtained were aligned and analyzed using BioEdit [14] and Mega 4 softwares [15]. The COI sequences of *W. bancrofti*from GenBank (Accession numbers CD455366.1 and AM 749236.1) and *Filaria martis* (GenBank Accession number AJ544880.1) were included in phylogenetic construction with the latter serving as the outgroup.

The test sequences were grouped into infrapopulations according to their collection sites. i.e. northern or southern. Aligned sequences were further analyzed in DnaSP v5 [16] for pairwise differences (p) and mutation rates (κ). Alignment positions were excluded after translation of sequences, resulting in 69 coding sites. We estimated the amount of variance within (infrapopulation) and among (interpopulation) populations using the AMOVA function in Arlequin v3.5.1.2 [17] with 20000 permutations. Population differentiation was measured with 100000 steps in Markov chain at 0.05 significance level.

The evolutionary history was inferred using the Maximum Parsimony method [18]. The percentage of replicate trees in which the associated taxa clustered together in the bootstrap test (5000 replicates) is shown next to the branches [19]. The MP tree was obtained using the Close-Neighbor-Interchange algorithm [20] in which the initial trees were obtained with the random addition of sequences (10 replicates).

## Results

Seven samples from Gomoa District (in the South) and 15 samples from Bongo District (in the North) were sequenced. The sequences have been submitted to GenBank with accession numbers GQ479497- GQ479518 in 2009. The similarity index between the Northern and Southern consensus sequences was calculated to be 38.3%. The mean evolutionary diversity between the two groups was 0.314 (SE = 0.028) while the mean evolutionary diversity for the entire population was 0.363 (SE = 0.038). The mean composition distance between the Northern samples was 0.090 while that between the Southern samples was 2.817. The mean composition distance between the two populations was 0.704. In addition, we computed the mutation frequency between the populations (Figure 1). The results revealed higher mutation frequencies in the southern population, compared to the northern population.

**Figure 2:**
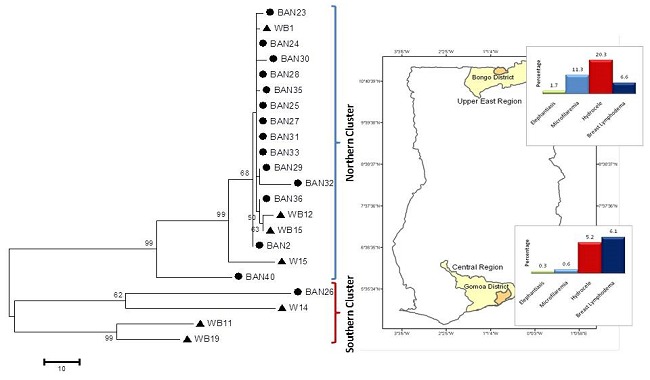
Maximum parsimony tree of COI sequences. Triangles denote samples from Gomoa in the South and circles denote samples from Bongo in the North. The map shows the sample sites and the regional disease prevalence data extracted from Gyapong et al, 1996.

**Figure 3:**
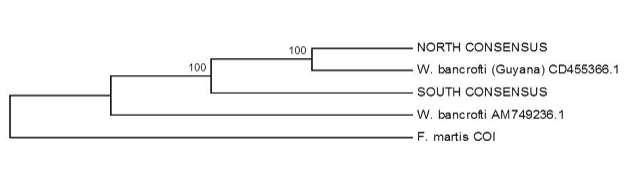
Maximum Parsimony tree of CO1 W. bancrofti consensus sequences from the Northern and Southern Ghana and other sequences from GenBank, using F. martis as an outgroup.

The evolutionary history was inferred using the Maximum Parsimony method [18]. The results of the MP tree show samples clustering according to the North and South classification (Figure 2). The rooted phylogenetic trees using *F. martis* as an outgroup (Figure 3) reveals the Southern samples to be the most distant population. 3 also shows the phylogenetic relation between the Ghanaian consensus sequences and other sequences from GenBank. The equality of evolutionary rate between the consensus sequences from the North and the South was tested using *F. martis* as an outgroup in Tajima′ relative rate test [21]. The X^2^ test statistic was 7.44 (P = 0.00637) at 0.05 confidence interval, rejecting the null hypothesis of equal rates between lineages.

**Figure 4:**
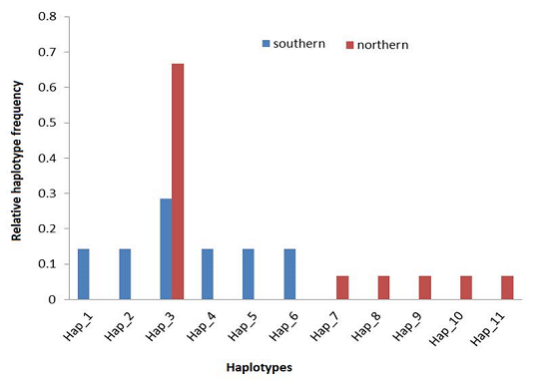
Relative frequencies of haplotypes.

Haplotype analyses revealed a total of 11 haplotypes (Hap) in all the 22 DNA sequences; 6 from the northern infrapopulation and 6 from the southern. A single haplotype (Hap_3) was shared between the two populations (Figure 4). Approximately 67% of the sequences in the northern population belonged to Hap_1, while each of the 4 sequences from the southern population was a different haplotype (Figure 4); suggesting high genetic variation within the southern samples.

**Figure 5:**
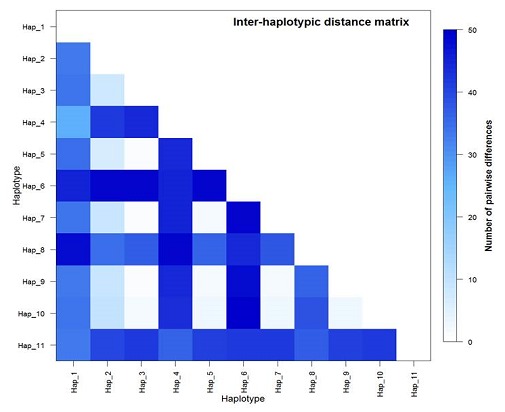
Distance matrix of interpopulation and intrapopulation haplotypic pairwise differences

Among the 69 loci used in the analyses, 58 polymorphic sites were observed between populations. The southern infrapopulation was more polymorphic than the northern infrapopulation (62 vrs 54 polymorphic sites). The measure of polymorphism is further emphasized by comparatively higher mean pairwise differences (π) in the southern population (30.81 +/- 15.37) than the northern (10.60 +/- 5.12). For example, for Hap_3 which is common to both populations, there seem to be a high number of pairwise differences (>20) when compared with the other haplotypes present in the southern intrapopulation (Hap_1- Hap_6) than in the northern intrapopulation (Hap_7-Hap_11) (Figure 5).

We tested the significance of these mutations in the two populations under the neutral theory. Under Tajima's test for selective neutrality, the southern population has a positive value (Tajima's D= 1.26, p= 0.852) while the northern population has a negative value (Tajima's D= -1.56, p= 0.038).

The AMOVA analysis showed that 87.2% of the variation observed is within population (intrapopulation) than between the populations (interpopulation). Therefore, the northern and southern populations are less genetically differentiated (Fixation index= 0.13, p= 0.03).

## Discussion

The data from this initial study indicates that the Southern samples are more distant. This data does provide relevant information about the possible route of entry of the parasite into Ghana and its subsequent spread. Consequently, because only anecdotal evidence of the parasites existence and prevalence in Ghana exists prior to 1990, this information is of great importance. This study, despite the small number of samples analyzed, does reveal some level of genetic variability in *W. bancrofti* in Ghana, thus adding to the very few studies that aimed at understanding the genetic heterogeneity of *W. bancrofti* [5, 6, 22].

The genetic differences observed in this study may be attributed to environmental selection pressures. This may explain the epidemiological and field observations of LF in Ghana. High prevalence of LF in Northern Ghana [3] could be as a result of a recent population expansion. Thus, the negative D value observed in the northern population (Tajima's D= -1.56), reveals that mutations have not resulted in high nucleotide diversity, based on the number of segregating sites. On the contrary, the positive D value (Tajima's D= 1.26) shows that the southern population may have suffered a population bottleneck, resulting in higher nucleotide diversity. From the phylogenetic tree the closeness of samples from both areas could be a result of migration of microfilaremic individuals from the South to the North or vice versa. Migration is an important routine activity in the Bongo District that occurs during specific times of the year, purposely for economic activities, and could result in the transfer of parasites from the South to the North.

The current LF control strategy is based on the assumption that MDA can be used alone in areas where *W. bancrofti* is transmitted by *Anopheles* species, including most endemic areas in Africa [23]. This strategy is dependent on the use of MDA with Albendazole and either DEC or Ivermectin to reduce circulating microfilariae below a threshold level, to break transmission by the disease vectors [24]. However, in other areas such as the Polynesian Islands of Moorea and Maupiti, over 50 years of MDA using DEC did not eliminate the disease [25]. We posit that the genetic differences in parasite population could be one of many possible explanations for this failure. These differences could be an indicator of selection pressure on the parasite suggesting that perhaps environmental selection pressures or other factors are in play. While no known cases of LF associated drug resistance in humans have yet been reported, the development of drug resistance is a realistic possibility as drug resistance to both Ivermectin and Albendazole is prevalent in nematodes of veterinary importance [26]. Also, there is some evidence of developing Ivermectin resistance in the closely related *O. volvulus* [27]. Furthermore, there have been reports of persistent residual Lf infections in some communities in Ghana and Burkina Faso, despite 5-8 rounds of treatments [28]. Whiles non-compliance and the influence by vector species may be attributed to these residual LF infections [24]; the possibilities of sub-optimal treatment responses to Ivermectin + Albendazole still remain. Thus, an understanding of the genetic distinctness of various parasite populations could be a useful indicator in assessing and responding to the development of drug resistance in the future.

The findings of this study may also suggest that the geographic strains of *W. bancrofti* can make a vector a more or less efficient one. In a susceptibility study [29], *Culex quinquefasciatus* strains from Liberia had a low susceptibility to *W. bancrofti* from Liberia, while the same strain had a high susceptibility to *W. bancrofti* from Sri Lanka. Consequently, the study concluded that the Liberian and Sri Lankan strains of *W. bancrofti* differed in their ability to infect specific mosquito strains and therefore confirms our suggestion that the geographic strain of *W. bancrofti* can improve vector competence. The same reasons may explain the different epidemiologies of LF in East and West Africa, as to why *Culex quinquefasciatus* is not a vector of LF in West Africa, but is a very efficient very in urban areas in East Africa [28, 30]. However, this needs to be further investigated.

In order to further explore the results produced in this study, we recommended that further studies with a much larger sample size and more detailed, population analyses be conducted on the diversity of *W. bancrofti* populations. This will enable the determination of profound conclusions on *W. bancrofti* diversity. The availability of the complete mitochondrial genome sequence of *W. bancrofti* [31] will enhance a further understanding of phylogenetic and geographic relationships between isolates, and assessing population diversity within endemic regions. Studies encompassing parasites from different geo-climatic regions will further enhance our understanding of *W. bancrofti* diversity as well as vector-parasite co-adaptations. Until more information, regarding the impact of *W. bancrofti* diversity on disease and treatment outcomes, becomes available, it is recommended that MDA be supplemented with vector control in order to ensure effective control of the parasite, in working towards the LF elimination goals of 2020.

## Conclusion

This study showed that there is considerable genetic variability within *W. bancrofti* populations in Ghana, differences that might explain the observed epidemiology of LF. Further studies are however required for an in-depth understanding of LF epidemiology and control.
